# 1402. The Landscape of Infections Caused by Rare Bacterial Pathogens

**DOI:** 10.1093/ofid/ofad500.1239

**Published:** 2023-11-27

**Authors:** Tahsin Farid, Reema Charles, Keyla Tumas, Heather Stone, Raghavendra Tirupathi

**Affiliations:** US Food & Drug Administration, Richmond, Texas; FDA, Silver Spring, Maryland; NIH/FDA, Washington, District of Columbia; FDA, Silver Spring, Maryland; Keystone Health, Chambersberg, Pennsylvania

## Abstract

**Background:**

Rare infectious diseases (RIDs) are a significant source of morbidity and mortality^1^. Most lack approved treatments and it is difficult to perform comprehensive trials on therapeutic efficacy due to their sporadic nature and distribution in resource limited settings^2^. Lack of monetary incentives has also discouraged drug development^3^. RIDs therefore represent an area of high unmet medical need. Here the landscape of rare bacterial infections (RBIs) is reviewed, as a pilot project, to explore the use of real-world data to better inform therapy.

Disclaimers and Affiliations

**Figure:**
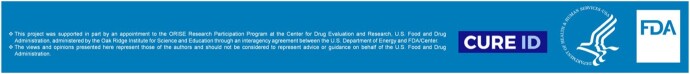

**Table 1:**
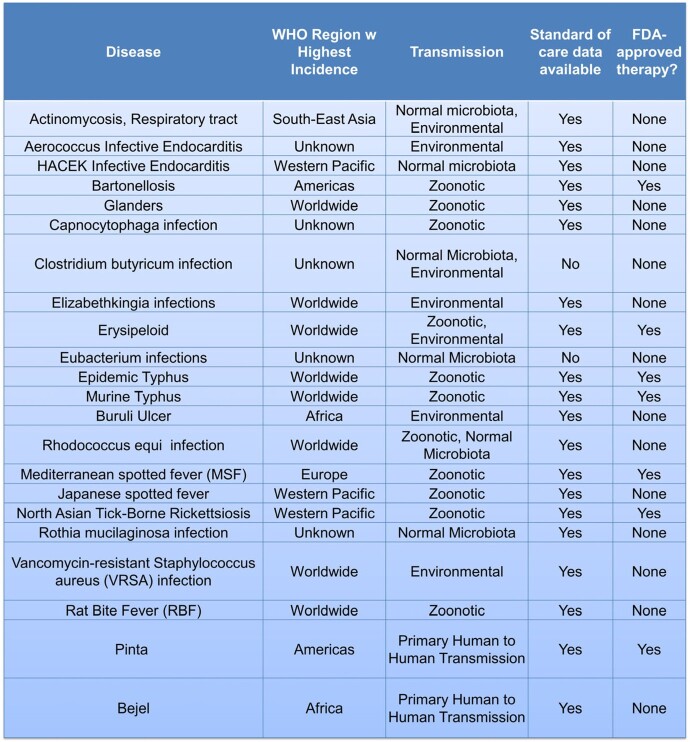
List of Included Rare Bacterial Infections

**Methods:**

A list of bacterial infections was curated by combining reportable infections from health agencies^4-7^ with the CURE ID^8^ database of diseases. This list was independently reviewed by two experts in infectious diseases to identify potential RBIs. A literature review identified which of these bacteria met the inclusion criteria: a global incidence of less than or equal to 10,000 cases per year (Figure 1). Data on each RBI that met the inclusion criteria was collected (Table 1) and analyzed.

**Figure 1.**
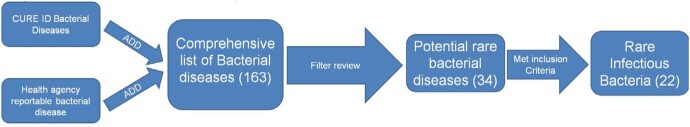
Data Flow Diagram

**Results:**

From the comprehensive list of 163 bacterial infections, 22 met the inclusion criteria. Figure 2 displays the distribution of World Health Organization (WHO) Regions where each RBI has the highest incidence. The most frequent mode of transmission amongst analyzed RBIs was zoonotic (Figure 3). 13 RBIs did not have FDA-approved therapies, but only 2 lacked a standard of care therapy (Table 2). Figure 4 depicts the transmission mode of RBIs according to the WHO region where they are most frequent.

**Figure 2:**
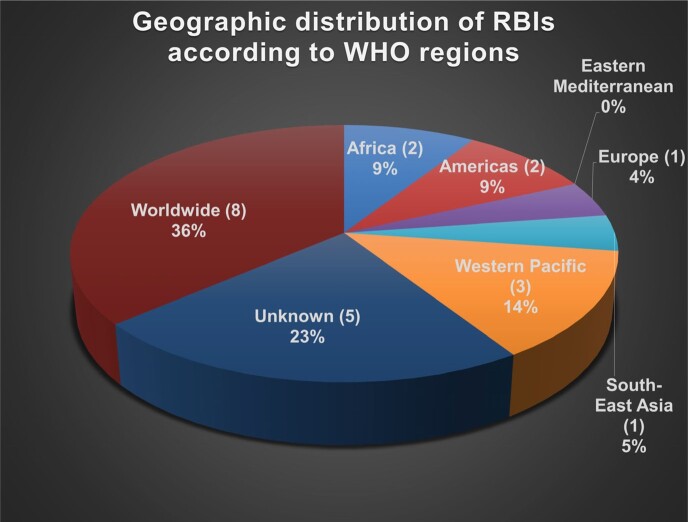
Percentage and number of diseases listed in table 1 by highest Incidence occurring per WHO Region

**Figure 3:**
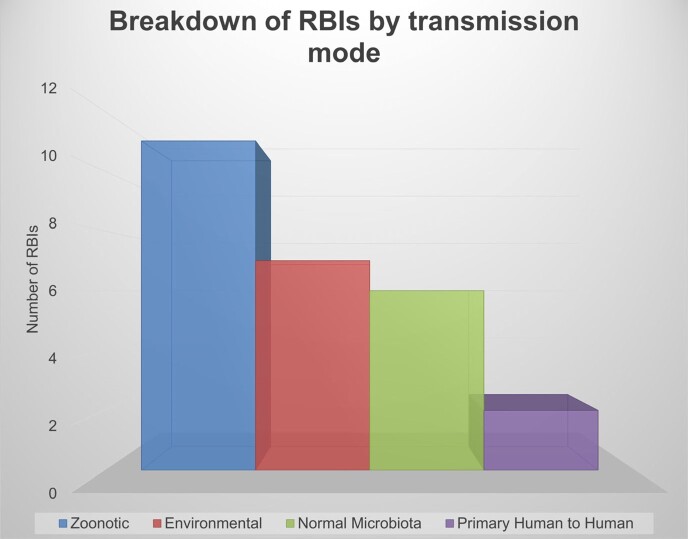
Breakdown of diseases in table 1 by mode of transmission

**Table 2:**
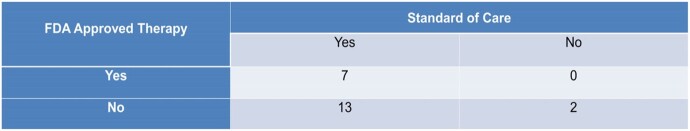
Standard of care availability plotted against FDA approved therapy availability

**Conclusion:**

It was expected that a majority of RIDs would occur through zoonoses while environmental and commensal organisms cause opportunistic infections in the immunocompromised. The majority of zoonoses had worldwide distributions. This does not imply higher incidence, but rather may indicate a lack of sustained endemicity with outbreaks in varied geographic areas when animal to human spillover occurs. Transmission patterns may also relate to socioeconomic conditions, climate change, war/civil unrest, and population growth. The lack of FDA-approved therapies for RIDs stems from the paucity of cases and little financial interest; real-world data may help improve treatment efficacy. This project aims to explore these possibilities and expand to all RIDs.

**Figure 4:**
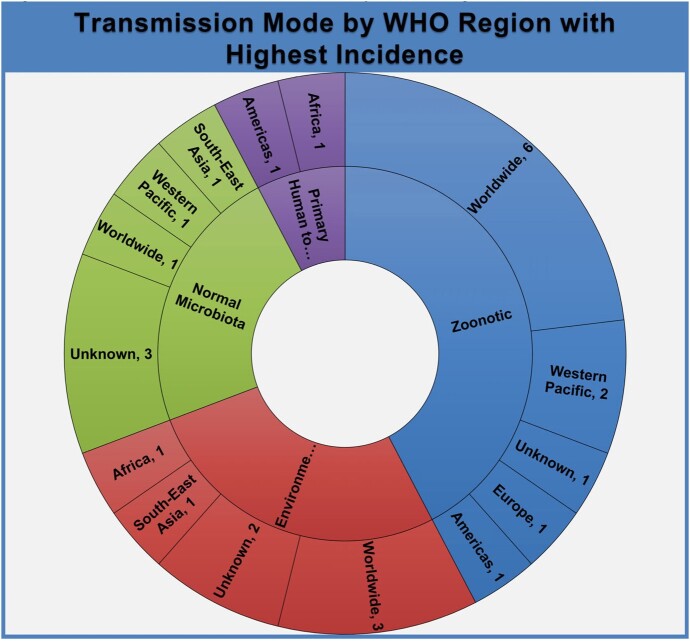
Plot of transmission mode by WHO region

References

**Figure:**
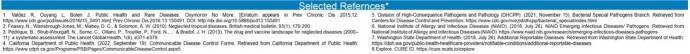

**Disclosures:**

**All Authors**: No reported disclosures

